# *Acacia hydaspica* R. Parker ameliorates cisplatin induced oxidative stress, DNA damage and morphological alterations in rat pulmonary tissue

**DOI:** 10.1186/s12906-018-2113-0

**Published:** 2018-02-02

**Authors:** Tayyaba Afsar, Suhail Razak, Ali Almajwal, Muhammad Rashid Khan

**Affiliations:** 1 Department of Biochemistry, Faculty of Biological Sciences, Quaid-i-Azam University, Islamabad, Pakistan; 2 Department of Animal Sciences, Faculty of Biological Sciences, Quaid-i-Azam University, Islamabad, Pakistan; 3 Department of Community Health Sciences, College of Applied Medical Sciences, King Saud University, Riyadh, Saudi Arabia

**Keywords:** DNA fragmentation, Pulmonary toxicity, Oxidative stress markers, Histopathological alterations

## Abstract

**Background:**

Cisplatin (CP) drug is platinum compounds used for the treatment of various human malignancies. However, adverse outcomes related to CP restrict its usage. *Acacia hydaspica* is a natural shrub with various pharmacological properties. The current investigation aimed to assess the protective potential of *A. hydaspica* polyphenol rich ethyl acetate extract (AHE) against cisplatin (CP) induced pulmonary toxicity.

**Methods:**

Rats were divided into six groups. Group 1 served as control (saline); Group 2 (drug control) recieved single dose of CP (7.5 mg/kg i.p.) on 1st day; Group 3 (extract control) (400 mg/kg bw, p.o.) received AHE for one week; Group 4 (Post-treated) and Group 5 (pretreated) received AHE (400 mg/kg bw/day, p.o) for 7 days after and before CP (7.5 mg/kg b.w., i.p.) respectively; Group 6 (Standard control) received silymarin (100 mg/kg b.w/7 days) before CP. At the end of dosing rats were sacrificed and pulmonary tissue samples were processed for the evaluation of antioxidant enzymes, oxidative stress markers, genotoxicity and histopathological alterations.

**Results:**

CP caused body weights loss and increase pulmonary tissue weight. The CP significantly increases oxidative stress markers and decreases tissue antioxidant enzyme levels. Furthermore, CP induced deleterious changes in the microanatomy of pulmonary tissue by rupturing the alveolar septa, thickening of alveolar walls, and injuring the cells with subsequent collapse of blood vessels. AHE pretreatment returned MDA, NO, H_2_O_2_ production and improved tissue antioxidant enzyme levels to near normalcy. The histological observations evidenced that AHE effectively rescues the lungs from CP-mediated oxidative damage. CP induction in rats also caused DNA fragmentation which was restored by AHE treatment. Our results suggest that pretreatment more significantly improve CP induced deleterious effects compared with post treatment indicating protective effect. Potency of AHE pretreatment is similar to silymarin.

**Conclusion:**

These findings demonstrated that *A. hydaspica* AHE extract might serve as potential adjuvant that prevents CP persuaded pulmonary toxicity due to its intrinsic antioxidant potential and polyphenolic constituents.

## Background

Adverse treatment reactions due to antineoplastic agents are a common form of iatrogenic injury [1], which prevails mostly in an idiosyncratic and indiscriminate manner. Pulmonary toxicity initiated by antineoplastic drugs is becoming a more frequently documented entity. Continuous exploitation of combined modality therapies increased the prevalence of secondary lung tumors and its multiplicity [2]. The pathological situation is usually nonspecific but some histological evidences help define the underlying agent [3]. Several pharmacological mediators employed in the cure of cancer have been associated with pulmonary toxicity. Mechanisms of impairment by these drugs consist of either direct pulmonary toxicity or indirect action via boosting inflammatory reactions. Risk factors for expansion of pulmonary impairments have been elucidated for a few agents but remain unclear for others. Chronic pneumonitis/fibrosis is the most common clinical feature for most of the categories of cytotoxic drugs [4].

Cisplatin (cis-dichlorodiammineplatinum (II) CP) is a synthetic anticancer drug generally used for the treatment of several human malignancies [1–3]. Whilst, therapeutic use of CP encouraged oxidative trauma and DNA damage in various non-cancerous tissues as well e.g. kidney, liver, testis, brain, lungs etc. [5]. CP and its different analogs are identified to mutilate DNA by making covalent adducts. This interface is seemingly liable for the organ damage including the lungs. Investigations in various rodent models verified the mutagenic and tumorigenic effects of CP. A single drug exposure produced skin and lung tumors in mice and rats, respectively [6]. CP is an effective therapeutic for lung cancer whilst free platinum intercalates or intracalates in DNA, induces severe injuriousness in experimental animals [2]. Cisplatin chemotherapy induced interstitial inflammation, fibrosis and structural lung damage associated with oblitrative bronchitis and increase perioperative complications in patients [7]. Pratibah and colleagues reported that CP administration for 18 weeks outcomes in pulmonary adenomas in A/J mice, although the mechanism behind this tumor promotion is not well understood. CP mediated pathogenesis of lung might be attributed to the reduced antioxidant defense, increase lipid peroxidation and ROS production [8]. The role of ROS in pulmonary impairment is further reinforced by the increased activity of free radical quenching enzymes in lungs confronted by a variety of toxins [9]. Despite various endeavors, the adverse effects associated with CP remains a key reason that confines its usage and potency in cancer therapy.

Treatment with antioxidants ameliorates or reduces the advancement of lung ailments i.e., in patients with COPD, lung cancer, asthma and acute respiratory distress syndrome (ARDS). Moreover, diet containing fruits and vegetables rich in flavonoids and other antioxidant compounds could be responsible for prevention of cancer and persuasive in ameliorating chemotherapeutic drugs prompted toxicity [10–12]. Adjuvant therapies that enhance the anti-tumor effects of cisplatin are actively being pursued. Globally the inquisitiveness for alternative and complementary medicines has gained much attention because of the chemo-preventive and therapeutic significance of medicinal plants in controlling of several oxidative stress-induced disorders [13–15]. Innumerable plant-derived metabolites like genistein, curcumin, resveratrol, indole-3-carbinol, epigallocatechin gallate (EGCG), and proanthocyanidin are capable to enhance efficacy and reduce harmful effects of traditional chemotherapeutic agents [16]. It is of essential requirements that therapeutic plants should be explored for their medicinal attributes; for the reason that most of the people in undeveloped countries’ practices alternative and complimentary medicines [17–19]. Genus *Acacia* possess species with diverse pharmacological properties, has come under extensive investigations in light of their anti-inflammatory, antitumor [20], antioxidant [21], wound healing [22], chemopreventive and antimutagenic [23] actions in various animal models [24]. *Acacia hydaspica* R. Parker synonym *A. eburnea* [25] belongs to family leguminosae is therapeutically important plant. The plant is normally used as fodder [25, 26] and is locally employed as antiseptic. In our previous lab experiments we revealed antioxidant, anti-cancer, anti-hemolytic, anti-inflammatory, antidepressant, and anxiolytic proficiencies of *A. hydaspica* [27–30]. Various bioactive metabolites were detected in *A. hydaspica* i.e., 1,2-Benzenedicarboxylic acid mono (2-ethylhexyl) ester, α-Amyrin, 2,6-dimethyl-N-(2-methyl-à-phenylbenzyl) aniline, Vitamin E, and Squalene, gallic acid, rutin, catechin, caffeic acid. Ethyl acetate fraction (AHE) of *A. hydaspica* showed excellent antioxidant activity in vitro. 7-*O*-galloyl catechin, +catechin and methyl gallate are the main bioactive metabolites with anticancer potential against breast and prostate cancer [27–29]. Various species of genus *Acacia* were reported for their antioxidant and protective potentials against lung toxicity in animal models [31]. *A. hydaspica* AHE fraction showed significant hepato-protective potential against cisplatin persuaded hepatic damage in rats [32]. Shahid and colleagues reported the protective potential of methanolic extract of *Acacia catechu* Willd bark. (MEBA) against the lung toxicity induced by B(a)P in mice. Pretreatment with MEBA at two different doses (200 and 400 mg/kg body weight) significantly ameliorates B(a)P-induced increased toxicity markers and activities of detoxifying enzymes along with the levels of glutathione content. It also significantly attenuated expression of apoptotic and inflammatory markers in the lungs and attenuated destruction of alveolar architecture and necrosis of the alveolar epithelium of the lungs [33]. *Acacia* honey ameliorated sodium arsenide persuaded oxidative trauma in the cardiac, pulmonary, and renal tissues of rats due to its antioxidant potential and polyphenol components [34]. Nikbakht et al. indicated that Gallic acid protect lungs from bleomycin induced increased in inflammatory or fibrotic changes, collagen content, levels of malondialdehyde (MDA), and pro-inflammatory cytokines such as TNF-α and IL1β. Furthermore Gallic acid reverse histological alterations and significantly increased non-enzymatic (total thiol) and enzymatic (glutathione peroxidase (GPx) antioxidant contents in the bleomycin treated rats’ lung tissue by its antioxidant properties [35]. Gallic acid inhibit oxidative damaged to DNA in lymphocytes, liver, colon and lungs of rats [36]. The development of secondary malignancy as a result of CP chemotherapy can be also prevented with polyphenol treatment as indicated by the study of Mimoto et al. illustrating that Epigallocatechin Gallate (ECGC) inhibit CP prompted pulmonary tumorigenesis and weight diminution in A/J mice [2, 37].

In view of protective potential of related *Acacia species* and polyphenolic compounds in animal models along with antioxidant and pharmacological activities of *A. hydaspica*. The present investigation aimed to find out the healing effect of AHE against CP-induced oxidative trauma and DNA injury in pulmonary tissue of rats via biochemical, DNA ladder assay and histopathological approaches.

## Methods

### Plant collection and preparation of AHE extract

*A. hydaspica a*erial parts were picked up from Kirpa charah area Islamabad, Pakistan. Plant sample was recognized by Dr. Sumaira Sahreen (Curator at Herbarium of Pakistan, Museum of Natural History, Islamabad) and voucher specimen (Accession No. 0642531) was submitted at the Herbarium of Pakistan, Museum of Natural History, Islamabad. *A. hydaspica* methanol extract was fractionated as previously described [30], and its ethyl acetate extract (AHE) (the fraction with maximum bioactivity was tested for protective proficiency against CP induced toxicity.

### Sample preparation

Cisplatin (CP) (Sigma-Aldrich, St. Louis, MO, U.S.A.) was diluted in saline to make accurate dose for testing. 7.5 mg/kg body weight dose of CP was selected on the basis of previous literature to induce acute renal toxicity [38]. Silymarin (100 mg/kg b.w) and AHE (400 mg/kg b.w) were prepared freshly in distilled water before treating.

### Acute toxicity evaluation

The acute toxicity study was conducted as per the guidelines 425 of Organization for Economic Cooperation and Development (OECD) for testing of chemicals for acute oral toxicity [39]. The detailed procedure is described in our earlier research [32]. General behavioral changes were detected by the previously described procedure [40]. Animals were observed continuously for 2 h and parameters which were observed were convulsion, tremor, aggression, excitation, loss of grasp, altered reactivity to touch, and sedation [41]. AHE was proved to be harmless at all tested quantities (up to 4000 mg/kg b.w) and it did not persuaded any noxious symptom in rats like sedation, convulsions, diarrhea and irritation. 400 mg/kg bw dose was selected for further in vivo evaluation.

### Experimental design

Thirty six male Sprague Dawley rats (200–225 g) were kept in the Primate Facility at Quaid-i-Azam University, Islamabad. The animals were placed in conventional steel cages at room temperature, fed with standard pellet diet and tap water under 12 h light/dark cycle at 25 ± 3 °C. Guidelines of national institute of animal health (NIH guidelines) were strictly adapted in order to conduct the experiment effectively. The experimental protocol (Bch#264) was approved ethical board of Quaid-i-Azam University, Islamabad. The investigational was planned according to earlier studies [42, 43] with trivial modifications. Animals were distributed into six groups (*n* = 6).

The following treatment procedure was adopted for the study.

*Group I*: Control; received distilled water.

*Group II*: CP treated; received single dose of CP (7.5 mg/kg b.w., i.p.).

*Group III*: AHE treated; received oral dose (400 mg/kg b.w) of AHE for 1 week.

*Group IV*: CP + AHE (Post-treated); administered AHE (400 mg/kg b.w/day, p.o.) for 1 week after CP injection [44].

*Group V*: AHE + CP (pretreated); received AHE (400 mg/kg body weight/day, p.o) for 1 week before CP (7.5 mg/kg b.w., i.p.).

*Group VI*: Sily+CP; received silymarin (100 mg/kg b.w., p.o) for 1 week before CP (7.5 mg/kg b.w., i.p.).

Male rats were chosen for study because they have persistent metabolism in contrast to females. Body weights of rats were note down at the start and completion of the experiment. The rats were humanly sacrificed by decapitation under light ether anesthesia. The lungs were removed, and rinsed with ice cold saline and dried with blotting paper, and weighted. Subsequently, half of the organs were preserved in liquid nitrogen and stored at − 80 °C for further enzymatic and DNA damage examination whereas the other portion was stored in 10% phosphate buffered formalin for histological analysis.

### Biochemical analysis

#### Homogenate preparation

Pulmonary tissue sample (100 mg) was homogenized in 10 volume of 100 mM KH_2_PO_4_ buffer including 1 mM EDTA, pH 7.4. The homogenate was centrifuged at 12000×g for 30 min at 4 °C and the supernatant was set aside in aliquots and put in storage at − 20 °C for examination of antioxidant enzymes, lipid peroxidation products, H_2_O_2_ and nitrite content.

#### Estimation of tissue protein content

The entire soluble proteins within the tissue homogenate were estimated as described earlier [45]. Tissue sample (100 mg) was homogenized in potassium phosphate buffer and centrifuged at 4 °C at 10000×g for 15–20 min to attain the supernatant. To the supernatant (0.1 ml) 1 ml of alkaline solution was poured and then vortexed. The mixture was incubated for 30 min and change in absorbance was observed at 595 nm. The concentration of protein in the sample was estimated using bovine serum albumin (BSA) standard calibration.

### Enzymatic antioxidant status

#### Catalase (CAT) activity

The CAT activity was estimated as described previously [46]. The CAT reaction solution consists of 35 μl homogenate, 625 μl of 50 mM of potassium phosphate buffer (pH 5), 100 μl of 5.9 mM H_2_O_2_. Change in the absorbance of the reaction solution was noted after 1 min at 240 nm. An absorbance change of 0.01 as units/min denotes one unit of catalase activity.

#### Peroxidase (POD) activity

POD enzyme activity was tested by previously defined procedure with slight modifications [46]. POD reaction solution contains 20 mM guaiacol (25 μl), 40 mM hydrogen peroxide (75 μl), 625 μl of 50 mM potassium phosphate buffer (pH 5.0) and 25 μl of tissue homogenate. Subsequently change in absorbance was determined at 470 nm after 1 min wait. One unit POD activity is equivalent to change in absorbance of 0.01 as units/min.

#### Superoxide dismutase (SOD) activity

The assessment of SOD activity was done by following Kakkar et al. method [47]. Tissue homogenate was centrifuge at 1500×g for 10 min and then at 10,000×g for 15 min. Supernatant was collected and 150 μl of supernatent was added to the aliquot containing 600 μl of 0.052 mM sodium pyrophosphate buffer (pH 7.0) and 186 mM of phenazine methosulphate (50 μl). To initiate enzymatic reaction 100 μl of 780 μM NADH was added. After 1 min, glacial acetic acid (500 μl) was added to stop the reaction. At 560 nm optical density was determined to enumerate the color intensity. Results were calculated in units/mg protein.

#### Quinone reductase assay (QR)

The Quinone reductase activity in pulmonary tissue samples was evaluated as described later [48]. Reaction mixture (3 ml) included 25 mM Tris-HCl buffer (2.13 ml; pH 7.4), 700 μl of BSA, 100 μl of FAD, 20 μl of 0.1 mM NADPH and 100 μl of pulmonary tissue homogenate. Reduction of dichlorophenolindophenol (DCPIP) was noted at 600 nm. Enzyme potency was estimated as nM of DCPIP reduced/min/mg protein by employing molar extinction coefficient of 2.1 × 10^4^/M/cm.

#### Reduced glutathione (GSH) assay

Reduced glutathione activity was tested as described by Jollow [49]. 500 μl of 4% sulfosalicylic acid was used for the precipitation of tissue homogenate. Subsequently 1 h of incubation at 4 °C all samples were centrifuged for 20 min at 1200×g. 33 μl supernatant was added to aliquots consisting of 900 μl of 0.1 M potassium phosphate buffer (pH 7.4) and 66 μl of 100 mM DTNB. Reaction of GSH with DTNB produced a yellow colored complex which was estimated at 412 nm. The GSH activity was presented as μM GSH/g tissue.

#### Glutathione-S-transferase (GST)

Scheme of Habig et al. [50] was applied for the assessment of GST. 150 μl aliquot of tissue homogenate was added to 720 μl of sodium phosphate buffer together with 100 μl of reduced glutathione (1 mM) and 12.5 μl of CDNB (1 mM). Optical density was recorded at 340 nm by spectrophotometer. GST activity was estimated through molar coefficient of 9.61 × 10^3^/M/cm as amount of CDNB conjugate formed per minute per mg protein.

#### Glutathione reductase assay (GR)

Glutathione reductase activity in tissue samples was examined as described by Carlberg and Mannervik [51]. The reaction reagent (2 ml) contained 1.65 ml phosphate buffer (0.1 M; pH 7.6), 100 μl EDTA (0.5 mM), 50 μl oxidized glutathione (1 mM), 100 μl NADPH (0.1 mM) and 100 μl of homogenate. Activity of enzyme was monitered by recording the absorbance of the vanishing of NADPH at 340 nm at 25 °C. Enzyme level was estimated as nM NADPH oxidized/min/mg protein by employing molar extinction coefficient of 6.22 × 10^3^/M/cm.

#### Glutathione peroxidase assay (GPx)

Glutathione peroxidase activity was calculated as defined earlier [52]. Entire volume of 2 ml reaction solution comprised of 1 mM EDTA (100 μl), 0.1 M phosphate buffer (1.49 ml; pH 7.4), 1 m M sodium azide (100 μl), 1 IU/ml glutathione reductase (50 μl), 1 mM GSH (50 μl), 0.2 mM NADPH (100 μl), 0.25 mM H_2_O_2_ (10 μl) and tissue homogenate (100 μl). The loss of NADPH was recorded at 340 nm at room temperature. Enzyme level was assessed as nM NADPH oxidized/min/mg protein employing 6.22 × 10^3^/M/cm molar extinction coefficient.

#### γ-Glutamyl transpeptidase (γ-GT)

The activity of γ-glutamyl transpeptidase was tested via Orlowski et al. scheme [53]. Reaction solution consist of an aliquot of 50 μl tissue homogenate, 250 μl of glutamyl nitroanilide (4 mM), 250 μl of glycyl glycine (40 mM) and 250 μl of MgCl2 (11 mM) which was primed with 185 mM Tris HCl buffer at room temperature. After 10 min of incubation, the reaction was stopped with the addition of 250 μl 25% trichloro acetic acid. Then centrifugation was done at 2500×g for 10 min and optical density was determined at 405 nm. Activity of γ-GT was determined as nM nitroaniline formed per min per mg protein by using molar extinction coefficient of 1.75 × 10^3^/M/cm.

### Oxidative stress markers

#### Lipid peroxidation assay (MDA content)

Protocol of Iqbal et al. [54] was implemented with trivial adjustments for the valuation of tissue lipid peroxidation. The reaction mixture consists of 0.1 M phosphate buffer of 290 μl (pH 7.4), 100 mM ferric chloride (10 μl), 100 mM ascorbic acid (100 μl) and 100 μl of homogenized sample. Incubation of the mixture was completed in shaking water bath for 1 h at 37 °C. Subsequently 10% trichloroacetic acid (500 μl) was added to stop the reaction. Afterwards 0.67% thiobarbituric acid (500 μl) was decanted and the reaction tubes were remained for 20 min in the water bath. Then all the reaction tubes were put on crushed ice bath for 5 min followed by centrifugation at 2500×g for 12–15 min. By spectrophotometer absorbance was calculated at 535 nm. By exploiting the molar extinction coefficient of 1.56 × 10^5^/M/cm. Outcomes were assessed as nM of TBARS generates per min per mg tissue at 37 °C.

#### Hydrogen peroxide assay

Hydrogen peroxide activity in tissue samples was examined by scheme described previously [55]. In the reaction mixture, 500 μl of 0.05 M phosphate buffer (pH 7), 100 μl of homogenate was added along with 100 μl of 0.28 nM phenol red solution, 250 μl of 5.5 nM dextrose and horse radish peroxidase (8.5 units) was added. Incubation was completed at room temperature for 60 min. 100 μl of NaOH (10 N) was added to stop the reaction. Then mixture tubes were centrifuged for 5–10 min at 800×g. The absorbance of the supernatant was calculated against reagent blank at 610 nm. Production of H_2_O_2_ was measured as nM H_2_O_2_/min/mg tissue by employing the standard curve of phenol red oxidized by H_2_O_2_.

#### Nitrite assay

Griess reagent was employed for the accomplishment of nitrite assay [56]. Briefly, tissue samples (100 mg each) were de-proteinised in 100 μl solution consist of 5% ZnSO_4_ and 0.3 M NaOH. Samples were Centrifuge at 6400×g for 15–20 min. 20 μl of supernatant was poured in a cuvette having 1 ml of Griess reagent, alteration in color was observed at 540 nm. Griess reagent 1 ml was used as a blank in the spectrophotometer (Smart Spec TM Spectrophotometer). Standard curve of sodium nitrite was utilized for the quantification nitrite concentration in tissues.

### DNA damage analysis

#### DNA fragmentation assay with diphenylamine reaction

Wu et al. [26] protocol was adopted for analysis of DNA fragmentation in pulmonary tissues. 100 mg tissue was homogenized in TTE solution. 100 μl of tissue homogenate (labeled B) was centrifuged at 200×g for 10 min at 4 °C and supernatant was collected and labeled S. subsequently the S tubes were centrifuged at 20,000×g at 4 °C for 10 min to separate intact chromatin and this was labeled as T. Next 1.0 ml of 25% TCA was pipetted in all tubes i.e., T, B, and S, and leave for overnight at 4 °C. After incubation samples were pelleted for 10 min at 18,000×g at 4 °C to recover the precipitated DNA. 160 μl of 5% TCA was added to each pellet and heated for 15 min at 90 °C then 320 μl of freshly made DPA solution was poured, vortexed and incubated for 4 h 37 °C. Optical density of samples was read at 600 nm with a spectrophotometer (Smart spec TM Plus, catalog # 170–2525).

#### DNA isolations and ladder assay

DNA isolation was performed by the methods of Wu et al. [26]. Tissue sample (100 mg) was placed in petri dishes and rinse with DNA Buffer and homogenized in 1 ml lysis buffer. Add 100 μl of proteinase K (10 mg/ml) and 240 μl 10% SDS in homogenized mixture and agitated slightly. Next the mixture was incubated overnight at 45 °C in a water bath then 0.4 ml of phenol was added and agitated for 5–10 min before centrifugation at 3000 rpm for 5 min at 10 °C. Supernatant was mixed with 1.2 ml phenol and 1.2 ml Chloroform/isoamyl alcohol (24:1) and shake for 5–10 min before centrifugation at 3000 rpm for 5 min at 10 °C. 25 μl of sodium acetate (pH 5.2, 3 M) and 5 ml ethanol was added to supernatant and agitated until DNA was precipitated. DNA was washed with 70% ethanol and then clean DNA was liquefied in TE buffer. The DNA concentration was checked at 260 and 280 nm. 5 μg of samples DNA and 0.5 μg DNA standard was loaded on 1.5% agarose gel containing ethidium bromide. Electrophoresis was performed for 45 min at 100 V batteries, and DNA was observed under digital gel doc system and photographed.

### Histopathological investigation

Pulmonary tissues from each group were fixed in a fixative containing absolute alcohol (85 ml), glacial acetic acid (5 ml) and 40% formaldehyde (10 ml). After dehydration tissue samples were fixed in parafilm to prepare blocks for microtomy. Tissues were sectioned 4–5 μm with microtome and stained with Hemotoxilin-Eosin (H&E) and inspected under light microscope (DIALUX 20 EB) at 40X.

#### Statistical analysis

Data are expressed mean ± SEM (*n* = 6). The statistical changes between different treatment groups was calculated by One way analysis of variance (ANOVA) followed by Tukey’s test using Graph pad prism 5 software. Level of significance was set at *p* < 0.05.

## Results

### Estimation of acute toxicity

AHE was revealed innocuous at all tested doses (up to 4000 mg/kg) and no appearance of any noxious symptom in rats like sedation, convulsions, diarrhea, and irritation was noticed. Throughout the 7 days of the valuation, no mortality was observed. Therefore, one tenth of the maximum dose, 400 mg/kg b.w. was used for the in vivo valuation of AHE.

### General toxicity

The rats treated with cisplatin, AHE alone or cisplatin+ AHE did not show any clinical signs of toxicity (such as unusual salivation, flicking movements, shiver, head and forelimb clonuses, spasms, incoordination, diarrhea, increased diuresis). No death was witnessed in both treated and untreated groups of rats during the experimental period.

### Body and organ weights

The effect of AHE treatment before and after CP intoxication on the body and Lung tissue weight is reported in Table 1. Data revealed significant (*p* < 0.001) fall in the final body weights of CP alone treated rats in comparison to both control and AHE treatment groups. Both pre and post treatments with AHE results in significant elevation in the final body weight of CP treated animals, and pretreatment seems to be more effective (*p* < 0.001) in reducing the growth impeding effects of CP. Results indicated significant difference in the lung weights of CP treated rats and control rats. AHE treatment significantly ameliorated lung weight fluctuations in contrast to CP alone treated group.

**Table 1:** Effect of cisplatin and/or AHE treatment on body weight and lung weight of rats

Group	Body weight (g)		Lung weight
	Initial	Final	
Control	218.3 ± 0.882	259.7 ± 0.667^b^	0.709 ± 0.01^b^
CP	220.0 ± 0.577	226.3 ± 0.66^a^	0.971 ± 0.03^a^
AHE alone	218.0 ± 0.577	258.3 ± 0.667^b^	0.701 ± 0.012^b^
CP + AHE	220.3 ± 0.881	244.0 ± 0.577^a,b^	0.839 ± 0.021^a**,b**,c*^
AHE + CP	219.0 ± 0.576	254.7 ± 0.881^a,b,c^	0.735 ± 0.015^b^
CP + Sily	218.7 ± 0.667	253.7 ± 0.667^a,b^	0.737 ± 0.022^b^

Data expressed as mean ± SEM (*n* = 6). a: significant difference of final body weight of group Vs. Control group at *p* < 0.001, b: significant difference of final body weight of group Vs. Cp-treated group at *p* < 0.001, c: significant difference of final body weight of AHE + CP pre-treated group Vs. CP + AHE post-treated group at *p* < 0.001. Sily; Silymarin. * or ** indicates significant difference at p < 0.05 and p < 0.001 respectively

### Protective effect of AHE on pulmonary enzymatic antioxidant status

The protective effect of AHE against CP induced alterations in POD, SOD, CAT and QR, are shown in the Table 2. CP inoculation markedly (*p* < 0.0001) declined the tissue levels of SOD, POD, CAT and QR. Post and pre-treatment of AHE significantly attenuated the suppressed enzyme activity in CP injected groups as compared to the only CP treated group. Post-treatment with AHE did not enhance the activity of POD enzyme when compared to CP alone treated group while, significant increase in activity of SOD, CAT and QR was noticed. The Pre-treatment of rats with AHE before CP intoxication seems to be more effective in restoring the above mention enzymes levels as compared to post-treatment group.

Table 3 shows the protective effect of AHE against CP-induced deterioration on GSH, GR, GST, γ-GT and GPx profile in pulmonary tissues. Cisplatin single dose significantly decreased the levels of phase II antioxidant enzymes in comparison to control. Data indicates that AHE pre-treatment and silymarin completely restored the GSH levels similar to control. Pre administration of AHE before CP intoxication bring about more significant (*p* < 0.001) augmentation in GSH, GR, GST, γ-GT and GPx levels as compared to AHE post administration, indicating the protective effect of AHE against CP induced deteriorations. The protective effect of AHE pre-treatment against CP provoked pulmonary toxicity was comparable to silymarin. AHE when orally administered alone, showed non-significant change in the level of above mentioned antioxidant enzymes compared to control. The protective effect of AHE may be mediated via modulation of enzyme systems via reducing the generation of free radicals.

**Table 2:** Effect of cisplatin (CP) and different treatments of AHE on lungs tissue antioxidant enzymes and GSH profile

Group	GSH (μM/g tissue)	GR (nM/min/mg protein)	GST (nM/min/mg protein)	γ-GT (nM/min/mg Protein)	GPx (nM/min/mg Protein)
Control	16.12 ± 0.578^b^	143.7 ± 1.342^b^	98.85 ± 0.918^b^	295.4 ± 1.113^b^	107.4 ± 0.730^b^
CP	8.334 ± 0.356^a^	98.02 ± 0.619^a^	68.17 ± 0.962^a^	82.82 ± 0.958^a^	54.08 ± 0.909^a^
AHE alone	6.38 ± 0.207^b^	144.0 ± 1.492^b^	99.79 ± 1.865 ^b^	295.6 ± 0.599^b^	108.8 ± 1.216^b^
CP + AHE	11.99 ± 0.305^a,b,d^	116.9 ± 0.813^a,b,d^	78.34 ± 1.076^a,b**,d**^	137.8 ± 1.017^a,b,d^	71.28.8 ± 0.501^a,b,c^
AHE + CP	15.63 ± 0.532^b,c^	135.0 ± 0.393 ^a,b,c^	89.65 ± 1.49 ^a**,b,c^	261.4 ± 0.802^a,b,c^	92.78 ± 1.216^a,b,c^
CP + Sily	15.29 ± 0.312^b^	133.8 ± 1.25^a,b^	87.60 ± 1.644^a,b^	264.3 ± 1.067^a,b^	95.64 ± 1.573^a,b^

Values expressed as mean ± SEM. a: Significance at *p* < 0.0001 Vs. control group, b: Significance at *p* < 0.0001 Vs. Cisplatin (CP) group. c: Significance at *p* < 0.0001 of AHE + CP pre-treated group Vs. CP + AHE post-treated group. d: Significance at *p* < 0.0001 of CP + AHE treatment groups Vs CP + Sily group. ^*^, ^**^: Significant difference at *p *< 0.05 and *p* < 0.001 respectively. Non-significant difference (*p* > 0.05) was recorded between control and AHE alone treated group in all parameters (One way ANOVA followed by Tukey’s multiple comparison tests)

### Protective effect of AHE against CP induced oxidative stress and lipid peroxidation

Oxidative stress owing to shift in the balance between oxidants and antioxidants in support of oxidants via decreasing the antioxidant capacity. H_2_O_2_ and nitrite (NO) level are considered to be an important indicator of oxidative stress. Malonyldialdehyde (MDA) is being one of the overwhelming products resulting from lipid peroxidation with recognized effect on the level of the human genome and is considered as clastogenic and genotoxic agent. Hence the amount of MDA in the biological sample can be measured as a sign of increased lipid peroxidation, therefore, an indicator of oxidative injury in vivo*.* The effect of AHE and CP on lung tissue protein, H_2_O_2_, NO and lipid peroxidation product (MDA) formation is given in the Table 4. Protein content in CP alone treated group decreased significantly; AHE post and pre-treatments ameliorated the toxic effect of CP. AHE pre-treatment restored the protein content relative to the control group, and enhanced (*p* < 0.001) protein content relative to AHE post-treatment group.

**Table 3:** Effect of cisplatin (CP) and different treatments of AHE on lung tissue protein, oxidative stress markers and lipid peroxidation

Group	Protein (μg/mg Tissue)	H_2_O_2_ (nM/min/mg Tissue)	Nitrite (content μM/ml)	MDA (nM/min/mg protein)
Control	1.722 ± 0.042^b^	2.192 ± 0.025^b^	44.59 ± 0.609^b^	3.66 ± 0.265^b^
CP	0.965 ± 0.072^a^	5.752 ± 0.081^a^	82.16 ± 1.531^a^	8.649 ± 0.403^a^
AHE lone	1.711 ± 0.056^b^	2.148 ± 0.034^b^	44.26 ± 0.385^b^	3.599 ± 0.245^b^
CP + AHE	1.237 ± 0.025^a,b*,d**^	4.498 ± 0.073^a,b,d^	68.14 ± 0.81^a,b,d^	6.965 ± 0.214^a,b**,d^
AHE + CP	1.582 ± 0.040^b,c**^	3.049 ± 0.136^a,b,c^	50.53 ± 0.410^a**,b,c^	4.614 ± 0.184^b,c^
CP + Sily	1.591 ± 0.075^b^	2.99 ± 0.012^a,b^	50.03 ± 1.149^a*,b^	4.396 ± 0.208^b^

Values expressed as mean ± SEM. a: Significance at *p* < 0.0001 Vs. control group, b: Significance at *p* < 0.0001 Vs. Cisplatin (CP) group. c: Significance at *p* < 0.0001 of AHE + CP pre-treated group Vs. CP + AHE post-treated group. d: Significance at *p* < 0.0001 of CP + AHE treatment groups Vs CP + Sily group. ^*^, ^**^: Significant difference at *p *< 0.05 and *p* < 0.001 respectively. Non-significant difference (*p* > 0.05) was recorded between control and AHE alone treated group in all parameters (One way ANOVA followed by Tukey’s multiple comparison tests)

**Table 4:** Effect of cisplatin (CP) and different treatments of AHE on lung tissue protein, oxidative stress markers and lipid peroxidation

Group	Protein (μg/mg Tissue)	H_2_O_2_ (nM/min/mg Tissue)	Nitrite (content μM/ml)	MDA (nM/min/mg protein)
Control	1.722 ± 0.042^b^	2.192 ± 0.025^b^	44.59 ± 0.609^b^	3.66 ± 0.265^b^
CP	0.965 ± 0.072^a^	5.752 ± 0.081^a^	82.16 ± 1.531^a^	8.649 ± 0.403^a^
AHE lone	1.711 ± 0.056^b^	2.148 ± 0.034^b^	44.26 ± 0.385^b^	3.599 ± 0.245^b^
CP + AHE	1.237 ± 0.025^a,b*,d**^	4.498 ± 0.073^a,b,d^	68.14 ± 0.81^a,b,d^	6.965 ± 0.214^a,b**,d^
AHE + CP	1.582 ± 0.040^b,c**^	3.049 ± 0.136^a,b,c^	50.53 ± 0.410^a**,b,c^	4.614 ± 0.184^b,c^
CP + Sily	1.591 ± 0.075^b^	2.99 ± 0.012^a,b^	50.03 ± 1.149^a*,b^	4.396 ± 0.208^b^

Values expressed as mean ± SEM. a: Significance at *p* < 0.0001 Vs. control group, b: Significance at *p* < 0.0001 Vs. Cisplatin (CP) group. c: Significance at *p* < 0.0001 of AHE + CP pre-treated group Vs. CP + AHE post-treated group. d: Significance at *p* < 0.0001 of CP + AHE treatment groups Vs CP + Sily group. ^*^, ^**^: Significant difference at *p* < 0.001. Non-significant difference (*p* > 0.05) was recorded between control and AHE alone treated group in all parameters (One way ANOVA followed by Tukey’s multiple comparison tests)

The oxidative stress markers (H_2_O_2_ and NO) were significantly raised in the CP treated group which were decreased significantly (*p* < 0.0001) with AHE pre-treatment. Similarly the MDA level was increased significantly (*p* < 0.0001) by CP treatment and diminished considerably (*p* < 0.001 and *p* < 0.0001) by both AHE post and Pre-treatments respectively, while pre-treatment was recorded to be more significant (*p* < 0.0001) in reducing the lipid peroxidation compared to post treatment group. AHE pre-treatment minimized the MDA content and the levels were similar to that of control and silymarin treated group. AHE treatment alone at 400 mg/kg b.w showed no alteration in protein content, oxidative stress markers and lipid peroxidation when compared to control.

### Molecular studies for analysis of DNA damage

#### Percent DNA fragmentation

**Fig. 1:**
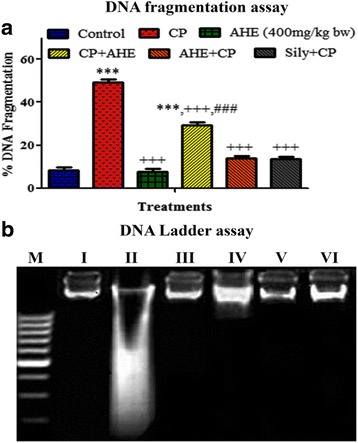
**a** DNA fragmentation (%) in different treatment groups, (**b**) Agarose gel showing DNA damage by CP and protective effects of Pre and post treatment of AHE and silymarin in pulmonary tissue. Lanes from left (M) low molecular weight marker, (I) control, (II) CP group, (III) AHE (400 mg/kg bw) group, (IV) CP + AHE group, (V) AHE + CP group, (VI) Sily + CP group

DNA fragmentation (%) showed marked changes in all experimental groups (Fig. 1 a). The AHE treated groups repaired the DNA damage induced by CP and decreased the DNA fragmentation showing the protective effects at the genetic level. Pre-administration of AHE reversed the level of DNA fragmentation (%) near to control group.

#### DNA ladder assay

DNA was extracted from the pulmonary tissue of the treated rats and different banding configurations were detected in Fig. 1 b. There was found much variation in the genomic DNA banding pattern from the pulmonary tissue of all experimental groups. In case of control group the genomic DNA gave single sharp band without degradation and tail pattern. CP treatment induced DNA damages in pulmonary tissues of rats which showed a peculiar type of continuous pattern of DNA fragmentation of up to 100 bp. AHE treatments showed marked repairing of the DNA damage. In the case of AHE + CP group there was more significant repairing of DNA damage compared to CP + AHE group; which showed partial protective effect (Fig. 1 b).

#### Histopathology assessment of lungs

**Fig. 2:**
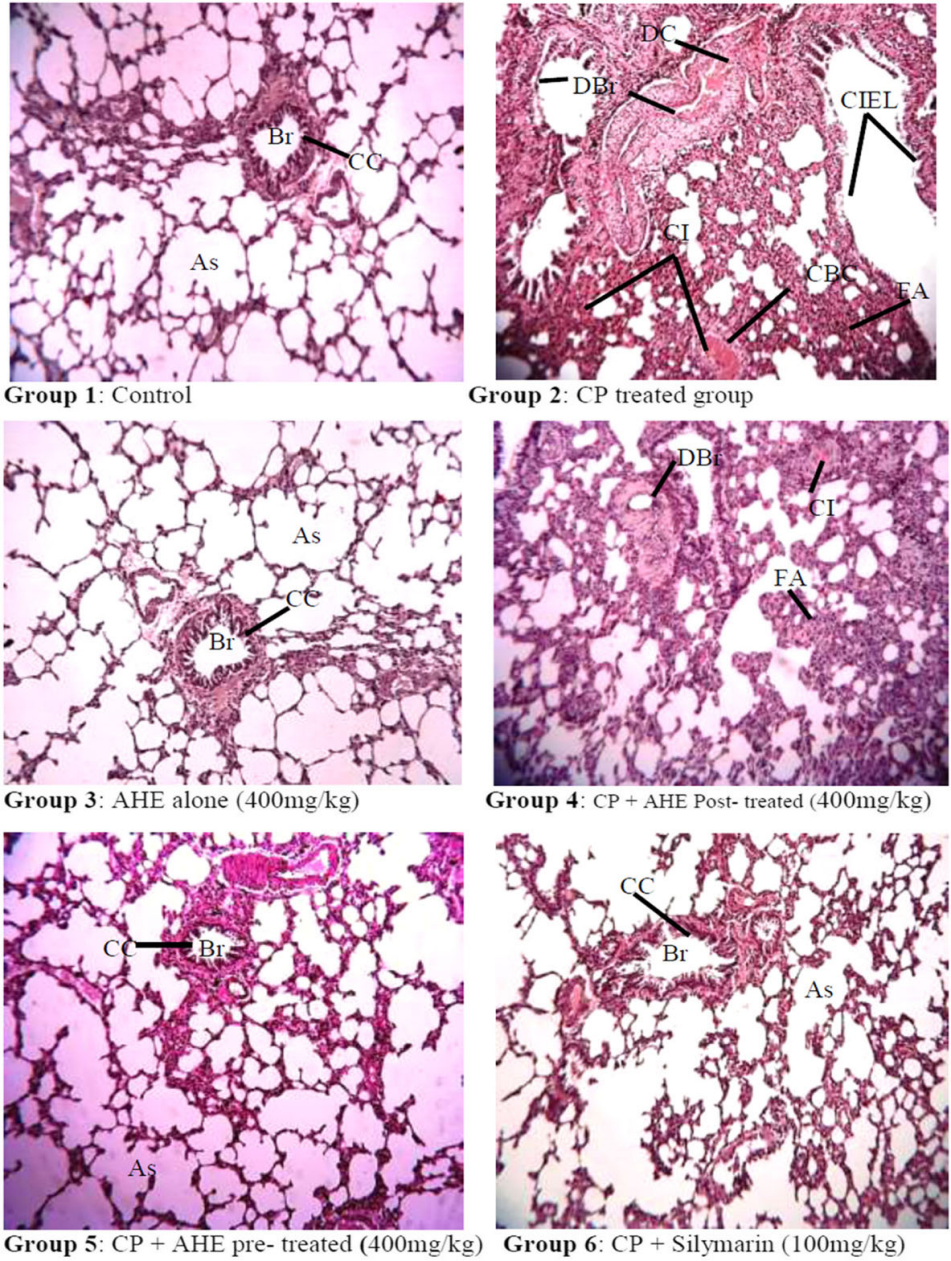
Histopathological effect of cisplatin and protective effect of AHE in rat Lungs. (H&E staining; magnification 40X). Group 1: Lung tissue section from control rats showing normal morphology. Group 2: tissue sections from CP-treated rats show degenerative changes, atrophy and cellular infiltrations. Group 3: Represents lung section from AHE alone treated rats. Group 4: AHE Post-treatment showed mild disorganizations, cellular infiltrations and fibroblast aggregations. Group 5: AHE Pre-treatment showed significant protection against CP-induced lung injury. Group 6: Showed protective effect of Silymarin treatment. AHE- *A. hydaspica* ethyl acetate fraction, CP-Cisplatin, Br-Bronchioles, As-Alveolar septum, CC-Clara cells, T- Tubules, CI- Cellular infiltrations, DBr-Disorganized bronchioles, FA- fibroblast aggregations, CIEL-collapsed inner epithelial cells

Pulmonary sections of control and AHE alone treated rats revealed the archetypal lung morphology with distinct alveolar septum, alveolar bronchioles, normal pear shape Clara cells, fibroblast, and type I and type II pneumocytes (Fig. 2). CP treatment induced severe degenerations including inflammatory cell infiltrations, alterations in alveolar septum, disruption of connective tissue and elastic fibers, blood vessel congestion, pulmonary edema, disorganized inner epithelium of alveolar bronchioles, fibroblast aggregations and interstitial hemorrhages. AHE + CP and Sily + CP treatment groups showed significant protection of pulmonary tissue against the CP side effects, and preserve morphology toward the control group. Administration of AHE after CP intoxication showed less protection as compared to its administration before the CP. The histopathological outcomes were in conformity with the results of pulmonary antioxidant enzymes, oxidative stress, lipid peroxidation status and DNA damage analysis.

## Discussion

Numerous drugs and chemotherapies employed in the treatment of various cancers have been associated with pulmonary toxicity [4]. Boosted ROS/RNS assembly persuades frequent DNA damages, impedement of apoptosis and stimulation of proto-oncogenes by prompting various signal transduction pathways that lead to damage of both DNA and protein [57, 58]. For that reason, it is feasible that enduring inflammation encouraged generation of ROS/RNS in the lung could incline the individuals towards lung cancer. CP administration has been associated with chronic obstructive pulmonary ailment and pulmonary dysfunction in humans, resulting as a consequence of the oxidative stress and deterioration in body’s antioxidant status [59]. Therefore, the current investigation aimed at evaluating the protective potential of *A. hydaspica* AHE (polyphenolic rich fraction) extract against CP induced pulmonary toxicity.

A disproportion between antioxidants and oxidants has been regarded as underlying mechanism in the pathogenesis of lung diseases. The lung has heavy reliance on enzymatic antioxidants i.e., Catalase (CAT), superoxide dismutase (SOD), and glutathione peroxidase (GPx). Our investigation revealed that administration of a single dose of CP induced pulmonary injuriousness in rats through substantial changes in the lung tissue antioxidant status, possibly due to increased lipid peroxidation and ROS generation, which consequently induce oxidative trauma. In the lung tissue of the CP administered rats, oxidative stress markers (MDA, NO and H_2_O_2_) levels were significantly elevated while antioxidant enzymes (SOD, CAT, POD, QR, GPx, γ GT GR, GST and GSH) levels were markedly declined. The low GSH-Px activity could be directly explicated by the diminished GSH quantity, for the reason that GSH is a substrate and cofactor of GSH-Px enzyme. As a result, low GSH content implies low GSH-Px activity that may possibly create an amplified oxidative stress tendency. These observations supported the assumption that free radicals play a major role in CP induced pulmonary toxicity [60]. However, enhanced activity level of SOD, CAT, POD, QR, GPx, GR, γ GT, GST and GSH enzyme of lung tissues and significant decrease in lipid peroxidation, NO and H_2_O_2_ content with AHE treatment may depict shielding ability of AHE to eradicate reactive free radicals generated by CP induced toxicity. AHE pretreatment seems to be more effective as compared to its administration after injury, and results of pretreatment were comparable to respective silymarin treated groups. Silymarin is a mixture of flavonoid isomers was used as standard extract for comparison. The mechanism action of silymarin is inhibition of lipid peroxidation and enhancement of endogenous antioxidants via its antioxidative and anti-inflammatory potential. Our results were in line with the study of Ayaz and coworkers indicating that pretreatment with methanolic bark extract of *Acacia catechu* (400 mg/kg body weight) significantly ameliorates benzo(a)pyrene induced increased toxicity markers and activities of detoxifying enzymes along with the levels of glutathione content. The underlying mechanism is the attenuated expression of apoptotic and inflammatory markers in the lungs [33]. Polyphenolic in *A. hydaspica* AHE fraction might be responsible for the protective effect via diminishing oxidative stress and increasing the antioxidant enzyme status. AHE has been shown to be rich in 7-*O*-galloyl catechin, catechin and gallic acid; metabolites with well-recognized antioxidant properties [61]. Hassan and colleagues [62] revealed the preventive potential of catechin and apigenin against cisplatin-induced alteration in antioxidant enzymes levels and oxidative stress marker by restoring them to normal. Furthermore, previous studies indicate that Gallic acid significantly deteriorated the inflammatory or fibrotic changes, collagen content, levels of MDA, and pro-inflammatory cytokines such as TNF-α and IL1β in pulmonary tissue of bleomycin treated rats. Gallic acid attenuates oxidative damage and reversed histopathological alterations induced by bleomycin by its antioxidant properties [35]. Similarly catechin a major component in AHE has shown protective potential against various toxicants i.e. catechin ameliorates chlorpyrifos-induced lung toxicity by restoring the activity of mitochondrial complex I and ATP biosynthesis [63]. Catechin compound was also able to restore the imbalance in superoxide dismutase and catalase activities as well as the decrease in NO levels induced by amiodarone. Protein and lipid oxidative damage and cell death were reduced by catechin and epicatechin in amiodarone -treated lung cells [64]. Various other studies involving catechin-rich fractions from other plants have demonstrated antineoplastic, antioxidant and anti-inflammatory properties [65]. As a consequence, it is not surprising that AHE extract of *A. hydaspica* exhibit protective potential because of the presence of catechins and flavonoids.

In the lung tissues, genotoxicity is of two types; direct genotoxic effects like DNA damage and induction of mutation or indirect effect caused by enzymes like cytochrome P-450 oxidase, flavin monooxidases etc., that generates ROS in the cells which leads to damage of both DNA and protein [57]. Oxidative stress causes intensification in oxidative DNA damage underlying various degenerative diseases [39]. In current finding, elevated levels of DNA fragmentation in CP administered group indicated the DNA damage through generation of free radicals. Previous finding have also been described that CP induce oxidative stress and genotoxicity with creation of DNA adducts and genetic alterations in various organs for instance brain [66], testis [67], stomach and lungs [68] etc. Relatively lesser amount of DNA fragmentation in AHE pretreated group point toward the protective role of AHE for lung tissues that was further confirmed by DNA ladder assay.

Histopathological examination revealed disruption in the alveolar septa, overcrowding of the blood capillaries, and thickening of alveolar walls, increase in fibroblast number, dilation of clara cells toward the lumen of the bronchiole, narrow lumen and necrosis of the alveolar epithelium in CP treated group. The disturbed histological structure of pulmonary tissues may be liable to make clear the potential of oxidative stress induced by CP to intoxicate the pulmonary tissues. Oxidative trauma induced by CP might link with morphological deteriorations; as free radicals have the aptitude to harm nucleic acids, proteins, and lipids. Resultantly they outcome in deterioration of enzymatic activity, initiate DNA strand breaks, lipid peroxidation, initiate cellular damage and structural disruption [69]. Effects of CP might be analogous to that of bleomycin intoxication indicating serious collapsed alveoli, epithelial damage, considerably thick inter alveolar septa with those of fibroblasts, and inflammatory cells infiltration [70]. Ethyl acetate fraction (AHE) of *A. hydaspica* suppressed CP intoxication in pulmonary tissues as displayed by a clear decrease in interstitial infiltration and collagen content. Histopathological examination supports the biochemical findings as illustrated by noteworthy lessening in pulmonary damage when AHE was used as a pre-treatment agent before CP administration. Pretreatment maintained the normal histological characteristics of the pulmonary cells. This indicates that administration of AHE prior to CP impede the free radicals generation and prevent cellular damages. In CP treated rats lung tissues are more prone to in vivo infection and inflammation. Therefore, suppression of lung inflammations may be significant for protection against CP induced toxicity to lung tissues. Pulmonary inflammatory reactions and edema are definitely associated with working of lung, includes pulmonary tract stress and oxygenation index [71]. Suppression of reactive species generation inhibited inflammation of lungs and damage in different models. Groups administered with AHE demonstrated considerable reduction in number of alveolar macrophages to decrease of epithelial damage and inflammatory cellular infiltration induced by CP. Previous reports have also confirmed the beneficial effects of antioxidants in pulmonary fibrosis. Phytochemicals in AHE comprising of flavan-3-ols (7-*O*-galloyl catechin, catechin) and methyl gallate were potentially responsible for observed protection against oxidative damage. Bors et al. also revealed that flavan-3-ols and flavonols are predominant antioxidants due to the existence of a 3-OH connected with the catechol (3′, 4′-dihydroxyl) unit [72]. Furthermore a study by Uzun and coworkers [63] revealed that catechin compound has ability prevents lung fibrosis and lessens histopathological changes in chlorpyrifos-induced lung toxicity. Recently, Lim and his colleagues evaluated the pharmacological impact of a *Morus alba* total extract (flavonoid rich) on inflammation of airways and observed a noteworthy decline in the overall amount of inflammatory cells and production of inflammatory mediators viz. TNF-α, IL-6 and NO by lung macrophages [73].

### Limitations of the study

Before making a definite statement about the possible suitability of AHE as an assistant to CP medications; additional studies are prerequisite to be undertaken in order to explicate the mechanism at the molecular level. For this western blot analysis and immunohistochemistry would provide information regarding signaling pathway in the pathogenesis of lung damage and protection afforded by Plant extract. Moreover the effect of plant extract at different dose levels and cancer models will provide knowledge on whether the extract interferes with anticancer ability of CP or not. Further studies using isolated compounds also help to determine the exact mechanism of action and to better ascertain the active compounds, and to find out if the pretreatment mitigates/abrogates the anti-cancer efficacy of CP.

## Conclusion

The results here suggest that deleterious side effects of CP may be restricted by the administration of AHE as a pretreatment agent. The underlying mechanism of protection is augmentation of endogenous antioxidant defense, diminished oxidative stress and inhibition of DNA damage.

## Abbreviations

AHE: *Acacia hydaspica* ethyl-acetate extract
ARDS: Acute respiratory distress syndrome
CAT: Catalase
COPD: Chronic Obstructive Pulmonary Disease
CP: Cisplatin
EGCG: Epigallocatechin gallate
GPx: Glutathione peroxidase
H_2_O_2_: Hydrogen peroxide
IL: Interleukins
MDA: Malonyldialdehyde
NADPH: Nicotinamide adenine dinucleotide phosphate
NO: Nitric oxide
SOD: superoxide dismutase
TBARS: Thiobarbituric acid reactive substances
TNF: Tumor necrosis factor
