# Correction to: *Acacia hydaspica* R. Parker ameliorates cisplatin induced oxidative stress, DNA damage and morphological alterations in rat pulmonary tissue

**DOI:** 10.1186/s12906-019-2658-6

**Published:** 2019-11-07

**Authors:** Tayyaba Afsar, Suhail Razak, Ali Almajwal, Muhammad Rashid Khan

**Affiliations:** 1 Department of Biochemistry, Faculty of Biological Sciences, Quaid-i-Azam University, Islamabad, Pakistan; 2 Department of Animal Sciences, Faculty of Biological Sciences, Quaid-i-Azam University, Islamabad, Pakistan; 3 Department of Community Health Sciences, College of Applied Medical Sciences, King Saud University, Riyadh, Saudi Arabia

**Correction to: BMC Complement Altern Med (2018) 18:49**

**https://doi.org/10.1186/s12906-018-2113-0**

Following publication of the original article [1], the author reported that Tables 3 and 4 were incorrect due to a production error.

**Table 3:** Effect of cisplatin (CP) and different treatments of AHE on lung tissue antioxidant enzymes

Group	POD (U/min)	SOD (U/mg protein)	CAT (U/min)	QR (nM/min/mg protein)
Control	9.56 ± 0.635^b^	1.366 ± 0.038^b^	18.48 ± 0.058^b^	128.2 ± 0.81^b^
CP	5.03 ± 0.271^a^	0.765 ± 0.019^a^	10.83 ± 0.049^a^	83.99 ± 0.486^a^
AHE alone	10.29 ± 0.314^b^	1.358 ± 0.058^b^	18.57 ± 0.057^b^	128.9 ± 0.179^b^
CP + AHE	6.22 ± 0.128^a, d^	0.982 ± 0.035^a,b*,d**^	14.15 ± 0.083^a,b, d^	98.82 ± 1.232^a.b, d^
AHE + CP	9.18 ± 0.185^b, c^	1.255 ± 0.038^b,c**^	17.07 ± 0.026^a,b, c^	119.5 ± 1.283^a,b, c^
CP + Sily	9.20 ± 0.208^b^	1.262 ± 0.021^b^	17.14 ± 0.081^a^,^b^	119.9 ± 1.008^a,b^

Values expressed as mean ± SEM. ^a^ Significance at *p* < 0.0001 Vs. control group, ^b^ Significance at *p* < 0.0001 Vs. Cisplatin (CP) group. ^c^ Significance at *p* < 0.0001 of AHE + CP pre-treated group Vs. CP + AHE post-treated group. ^d^ Significance at *p* < 0.0001 of CP + AHE treatment groups Vs CP + Sily group. ^*^, ^**^: Significant difference at *p* < 0.001. Non-significant difference (*p* > 0.05) was recorded between control and AHE alone treated group in all parameters (One way ANOVA followed by Tukey’s multiple comparison tests)

**Table 4:** Effect of cisplatin (CP) and different treatments of AHE on lungs tissue antioxidant enzymes and GSH profile

Group	GSH (μM/g tissue)	GR (nM/min/mg protein)	GST (nM/min/mg protein)	γ-GT (nM/min/mg Protein)	GPx (nM/min/mg Protein)
Control	16.12 ± 0.578^b^	143.7 ± 1.342^b^	98.85 ± 0.918^b^	295.4 ± 1.113^b^	107.4 ± 0.730^b^
CP	8.334 ± 0.356^a^	98.02 ± 0.619^a^	68.17 ± 0.962^a^	82.82 ± 0.958^a^	54.08 ± 0.909^a^
AHE alone	6.38 ± 0.207^b^	144.0 ± 1.492^b^	99.79 ± 1.865 ^b^	295.6 ± 0.599^b^	108.8 ± 1.216^b^
CP + AHE	11.99 ± 0.305^a,b, d^	116.9 ± 0.813^a,b,d^	78.34 ± 1.076^a,b**,d**^	137.8 ± 1.017^a,b,d^	71.28.8 ± 0.501^a,b,c^
AHE + CP	15.63 ± 0.532^b, c^	135.0 ± 0.393 ^a,b,c^	89.65 ± 1.49 ^a**,b,c^	261.4 ± 0.802^a,b,c^	92.78 ± 1.216^a,b,c^
CP + Sily	15.29 ± 0.312^b^	133.8 ± 1.25 ^a,b^	87.60 ± 1.644^a,b^	264.3 ± 1.067^a,b^	95.64 ± 1.573^a,b^

Values expressed as mean ± SEM. ^a^ Significance at *p* < 0.0001 Vs. control group, ^b^ Significance at *p* < 0.0001 Vs. Cisplatin (CP) group. ^c^ Significance at *p* < 0.0001 of AHE + CP pre-treated group Vs. CP + AHE post-treated group. ^d^ Significance at *p* < 0.0001 of CP + AHE treatment groups Vs CP + Sily group. ^*^, ^**^: Significant difference at *p* < 0.001. Non-significant difference (*p* > 0.05) was recorded between control and AHE alone treated group in all parameters (One way ANOVA followed by Tukey’s multiple comparison tests)

The corrected tables are given below.
